# Dilute Hyaluronic Acid for Lipoatrophy: Three Cases and Literature Review

**DOI:** 10.7759/cureus.105227

**Published:** 2026-03-14

**Authors:** Juliana M O'Reilly, David S Kirwin, Travis Frantz, Willis Lyford

**Affiliations:** 1 Graduate Medical Education, Walter Reed National Military Medical Center, Bethesda, USA; 2 Dermatology, Naval Medical Center San Diego, San Diego, USA

**Keywords:** facial, hiv, hyaluronic acid, lipoatrophy, lipodystrophy, off-face, radiation, soft tissue filler, steroid, trauma

## Abstract

Localized lipoatrophy, characterized by subcutaneous fat loss, can result from trauma, injectable drugs, panniculitis, or idiopathic causes, leading to outcomes ranging from self-resolution to permanent disfigurement. This case series presents a novel approach using dilute hyaluronic acid (HA) for three cases of lipoatrophy (trauma-induced, radiation-induced, and steroid-induced), with all cases showing notable improvement after one session. This approach represents a promising, low-risk alternative for managing lipoatrophy of various etiologies, with potential benefits for patient quality of life.

## Introduction

Localized lipoatrophy is a form of subcutaneous fat loss. Acquired lipoatrophy is particularly rare, with known causes including trauma-induced, radiation-induced, injectable drug-induced, panniculitis-associated, and idiopathic causes [1-5]. Depending on the cause, the prognosis varies. Certain cases are self-limited, while others result in permanent disfigurement, which can lead to significant consequences for patients' self-image and quality of life [5].

Despite its clinical significance, there is limited consensus on optimal treatment for acquired lipoatrophy across different etiologies. Injectable fillers have been widely used for soft tissue restoration in both cosmetic and reconstructive settings. Hyaluronic acid (HA) fillers are biocompatible, viscoelastic hydrogels used for soft tissue augmentation. Their ability to bind to water and integrate into the extracellular matrix is a key mechanism by which they provide structural support [6].

Few minimally invasive, low-risk interventions have been described for acquired lipoatrophy. Most treatment options are invasive procedures, including fat transfer or grafting, and much of the literature focuses on HIV-associated facial lipoatrophy [1]. As a result, there is a gap in the literature regarding simple, reproducible techniques for treating lipoatrophy resulting from trauma, radiation, or corticosteroid injections. We present a novel approach using dilute HA to treat various causes of lipoatrophy, with dramatic improvement after only one session in three cases.

## Case presentation

We report three cases, with patient characteristics, treatment details, and outcomes summarized in Table 1.

**Table 1:** Dilute Hyaluronic Acid Filler Use in Lipoatrophy: Sessions, Volumes, Adverse Effects, and Follow-up HA: hyaluronic acid, KS: Kaposi sarcoma, HIV: human immunodeficiency virus, ILK: intralesional Kenalog, PLLA: poly-L-lactic acid.

Case	Etiology	Site	Total Sessions	Volume per Session (dilute HA)	Adverse Effects	Follow-up Plan
1	Radiation induced lipoatrophy (HIV + KS)	Face, site of prior KS radiation	3	08/2024: 2.0 cc; 10/2024: 1.55 cc; 11/2024: 1.5 cc	None	2-3 month follow-up; ongoing PLLA treatment; skin cancer surveillance
2	Steroid-induced lipoatrophy (post-ILK for hypertrophic scar)	Right forearm	2	08/2024: 2.0 cc; 10/2024: 0.6 cc	Tyndall effect (resolved by 4-week follow-up without intervention)	Follow-up as needed
3	Trauma-induced lipoatrophy	Left lateral leg	4	04/2024: 0.6 cc; 05/2024: 0.2 cc; 10/2024: 1.0 cc; 01/2025: 0.6 cc	None	Follow-up in 3-6 months as needed

Case 1

A 66-year-old man with a >30-year history of well-controlled HIV and prior Kaposi sarcoma (treated with radiation therapy more than two decades ago) presented for evaluation of a 2 × 2 cm hypopigmented, atrophic, depressed plaque on the left cheek, consistent with chronic radiation-induced changes and localized lipoatrophy. His medical history is also notable for multiple nonmelanoma skin cancers and colorectal carcinoma, previously managed with surgical resection and chemotherapy. After discussion of management options, including correction with dilute hyaluronic acid filler, the patient elected to proceed with treatment.

**Figure 1:**
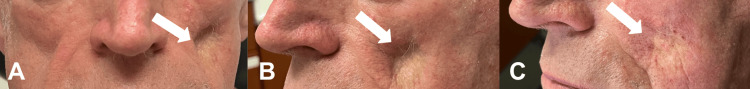
Case 1: Pre- and Post-injection of Dilute Hyaluronic Acid Left cheek before dilute HA injection (a,b) versus immediately after dilute HA injection (c) in a patient with radiation induced lipoatrophy.

A 2:1 dilution of HA with normal saline was prepared, and 2.0 cc was injected into the subdermal plane (Figure 1). At the six-week follow-up, significant cosmetic improvement was noted. The patient subsequently underwent two additional treatment sessions using the same 2:1 dilution. Continued improvement was noted without adverse effects. Following these sessions, the patient elected to transition to poly-L-lactic acid (PLLA) biostimulation for further management. He continues to follow closely with the dermatology clinic for skin cancer surveillance and plans to follow up as needed for filler treatments.

Case 2

A 28-year-old woman with a history of a punch biopsy-induced hypertrophic scar on the right forearm was treated with serial intralesional triamcinolone. The patient presented to dermatology with a 2.5 cm × 3.5 cm convex hypopigmented plaque and an overlying pink, round, soft scar, consistent with lipoatrophy secondary to steroid injection. Review of systems and other past medical history were noncontributory. After discussion of treatment options, along with risks, benefits, and alternatives, the patient decided to proceed with HA filler injection.

**Figure 2:**
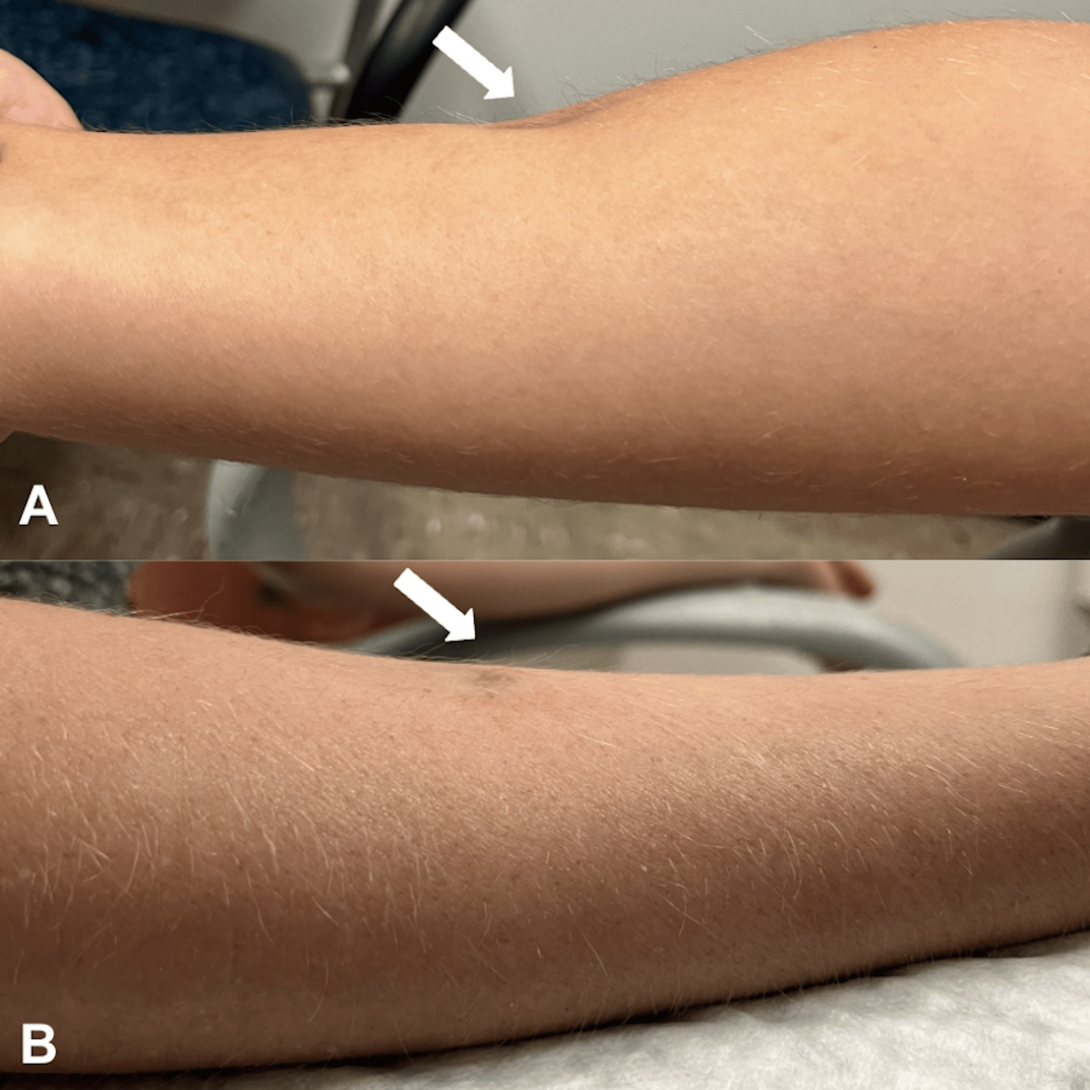
Case 2: Pre- and Post-injection of Dilute Hyaluronic Acid Right anterior forearm of a patient with steroid-induced lipoatrophy secondary to intralesional Kenalog injections for hypertrophic scar. (a) Medial view before dilute HA injection; (b) lateral view after dilute HA injection (same arm, different views).

A dilute HA preparation was made by a 2:1 dilution of normal saline and HA filler. Two cc of this dilute HA was injected into the subdermal plane during the initial session. Immediately following treatment, the treated area developed an ill-defined blue-purple hue consistent with a Tyndall effect (Figure 2). Reassurance was provided, as our experience with this procedure suggests that high-volume instillation in the deeper dermis/subcutis results in a temporary Tyndall effect, which resolves in three to five days following treatment. At the four-week follow-up, the blue-purple discoloration had resolved, and marked cosmetic improvement was observed. At this visit, the patient received a second dilute HA filler treatment (0.6 cc) and was recommended to return for follow-up as needed.

Case 3

A 33-year-old female presented for evaluation of lipoatrophy on her left lateral leg, secondary to a motorcycle accident two years prior. Review of systems and past medical history were noncontributory. After discussion of treatment options, along with risks, benefits, and alternatives, the patient decided to proceed with HA filler injection.

**Figure 3:**
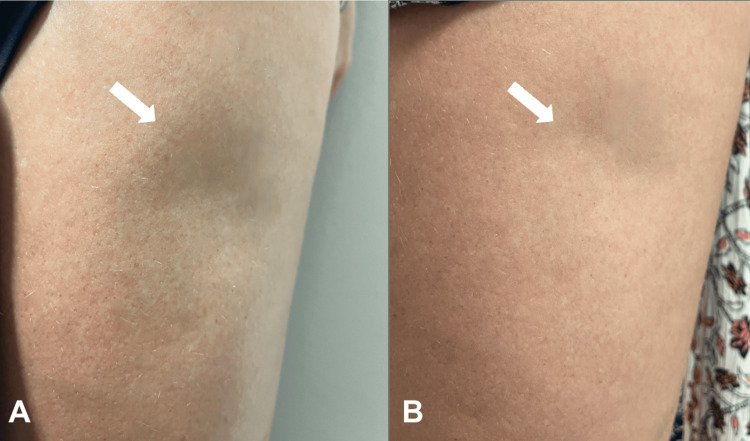
Case 3: Pre- and Post-injection of Dilute Hyaluronic Acid Left upper thigh before dilute HA injection (a) versus after dilute HA injection (b) in a patient with trauma induced lipoatrophy secondary to a motorcycle accident.

A dilute HA preparation was made by a 2:1 dilution of normal saline and HA filler, and 0.6 cc of dilute HA was injected into the subdermal plane during the initial session (Figure 3). The patient returned to the clinic five weeks later and reported a cosmetically pleasing response without any adverse effects. At her second treatment session, she received 0.2 cc of the same dilute HA filler. Five months later, a third session was performed using 1.0 cc. Nearly one year after the initial treatment, she underwent a fourth session with 0.6 cc. Across all sessions, she reported cosmetically satisfactory outcomes and experienced no adverse effects. She was instructed to follow up with dermatology as needed for continued soft tissue augmentation with dilute HA filler, given the excellent cosmetic response.

## Discussion

Lipoatrophy is a form of lipodystrophy that results in fat loss in the subcutaneous tissue layer. There are two main categories of lipoatrophy: congenital and acquired. Etiologies of acquired lipoatrophy include trauma, aging, HIV, radiation-induced causes, and connective tissue diseases such as lupus erythematosus profundus and morphea [1,2]. Causes of trauma-induced lipoatrophy include physical injury, repeated pressure, and injectable medications [1]. Though still rare, a well-documented type of trauma-induced lipoatrophy is lipoatrophia semicircularis, associated with specific workplace conditions such as desk height [3].

There are limited reports of trauma-induced lipoatrophy and its subsequent treatment. The few cases published have reported a range of outcomes and treatments. One case describing lipoatrophy secondary to transgluteal drainage of a suspected abscess reported spontaneous resolution after a four-month follow-up [4]. A case report describing the use of PLLA for trauma-induced facial atrophy reported improvement in volume overlying the zygomatic arches, cheeks, nasolabial folds, temples, and chin, as well as increased facial harmony after six months of treatment. The article also reported that the most dramatic improvement involved the patient's increased confidence and outlook on life [5].

HA use for the treatment of lipoatrophy has been described in various forms, including cases of morphological asymmetry, facial lipoatrophy, and treatment of cellulite [7,8]. There has been one case describing the use of large-particle HA gel in the treatment of radiation-induced lipoatrophy of the lower eyelid, but it is otherwise largely undescribed [2]. There are no reports of HA filler for steroid-induced lipoatrophy. The majority of cases describing treatment of steroid-induced lipoatrophy involve intralesional saline [9]. In our review, we found that the majority of published evidence describing the use of HA is for HIV-associated facial lipoatrophy treatment [10,11]. The lack of treatment options in the literature for these various causes of lipoatrophy highlights a need for further research and publication of successful management strategies.

In our case series, dilute HA provided sustained cosmetic improvement across multiple etiologies of lipoatrophy. Two patients continue to follow with the dermatology clinic on an as-needed basis for retreatment with dilute HA filler, reflecting durable response and patient satisfaction. One patient elected to transition to PLLA for ongoing volume restoration, underscoring the role of dilute HA as both a primary and bridging therapeutic option depending on patient goals and disease course. Potential complications associated with HA filler include granuloma formation, nodules, persistent Tyndall effect, and filler migration, which have been described in multiple reviews of dermal filler adverse effects [12-16]. Strategies to mitigate these risks include careful injection technique with appropriate depth and volume, consideration of hyaluronidase for correction of undesirable outcomes, and close clinical monitoring.

## Conclusions

In conclusion, we report a novel technique using dilute HA injection for the treatment of lipoatrophy of varying etiologies. Dilute HA has been shown to have improved tissue spread and tissue integration compared to undiluted formulations. However, optimal dilution parameters remain poorly defined, and potential limitations include decreased duration of effect and reduced lift. Importantly, the use of dilute HA may lower the risk of adverse events, including HA nodule formation, by reducing product concentration and improving homogeneous distribution within the tissue plane. Because the most serious complications of hyaluronic acid (HA) filler, such as angioedema, vascular compromise, and tissue ischemia, are closely associated with injection volume, product concentration, and inadvertent intravascular embolization, the use of a more dilute formulation may theoretically reduce the likelihood and severity of these adverse events. Large systematic reviews and consensus guidelines emphasize that complication rates are influenced by injection technique, product properties, and tissue behavior, all of which may be favorably impacted by dilution strategies.

At the time of this literature review, there are limited reports addressing the management of radiation-induced, steroid-induced, and trauma-induced lipoatrophy in general, and no cases describing a dilute HA filler technique for any of the above etiologies. We hope to bring awareness of dilute HA as a potential low-risk, semipermanent treatment modality to consider for patients with lipoatrophy.
